# Quantifying Surface‐Height Change Over a Periglacial Environment With ICESat‐2 Laser Altimetry

**DOI:** 10.1029/2020EA001538

**Published:** 2021-08-11

**Authors:** R. J. Michaelides, M. B. Bryant, M. R. Siegfried, A. A. Borsa

**Affiliations:** ^1^ Department of Geophysics Colorado School of Mines Golden CO USA; ^2^ Institute of Geophysics and Planetary Physics Scripps Institution of Oceanography University of California San Diego La Jolla CA USA

**Keywords:** ICESat‐2, altimetry, permafrost, InSAR

## Abstract

We use Ice, Cloud, and land Elevation Satellite 2 (ICESat‐2) laser altimetry crossovers and repeat tracks collected over the North Slope of Alaska to estimate ground surface‐height change due to the seasonal freezing and thawing of the active layer. We compare these measurements to a time series of surface deformation from Sentinel‐1 interferometric synthetic aperture radar (InSAR) and demonstrate agreement between these independent observations of surface deformation at broad spatial scales. We observe a relationship between ICESat‐2‐derived surface subsidence/uplift and changes in normalized accumulated degree days, which is consistent with the thermodynamically driven seasonal freezing and thawing of the active layer. Integrating ICESat‐2 crossover estimates of surface‐height change yields an annual time series of surface‐height change that is sensitive to changes in snow cover during spring and thawing of the active layer throughout spring and summer. Furthermore, this time series exhibits temporal correlation with independent reanalysis datasets of temperature and snow cover, as well as an InSAR‐derived time series. ICESat‐2‐derived surface‐height change estimates can be significantly affected by short length‐scale topographic gradients and changes in snow cover and snow depth. We discuss optimal strategies of post‐processing ICESat‐2 data for permafrost applications, as well as the future potential of joint ICESat‐2 and InSAR investigations of permafrost surface‐dynamics.

## Key Points

- ICESat‐2 altimetry can resolve surface subsidence that is related to changes in snow‐cover depth and seasonal thawing of the active layer
- ICESat‐2 measurements of surface‐height change are affected by along‐track topographic gradients and complex surface roughness
- Complementary ICESat‐2 and InSAR datasets can be jointly leveraged for future studies in periglacial environments

## 1 Introduction

Permafrost, defined as ground that remains frozen for two or more consecutive years, underlies 24% of the Northern Hemisphere and contains stores of bound carbon in the subsurface (primarily carbon dioxide and methane) amounting to 60% of the world's soil carbon (Turetsky et al., 2020). The Arctic, where the majority of permafrost is located, is the fastest‐changing component of the global climate system, with air temperatures across the Arctic currently increasing at roughly twice the average global rate (Jorgenson et al., 2001; Serreze & Barry, 2011). Rising air temperatures can increase the magnitude of seasonal thawing and freezing of the uppermost portion of the permafrost column (the “active layer”) and can induce permanent thawing and unrecoverable loss of permafrost. Both of these processes can result in decomposition of bound soil carbon and its release into the atmosphere (Natali et al., 2019). Results from the Coupled Model Intercomparison Project Phase 5 (CMIP5) suggest that global permafrost extent may decrease anywhere from 20%–37% by the end of the 21st century (Wang et al., 2019). As simultaneously one of the largest carbon reservoirs in the global carbon cycle and one of the fastest‐warming regions on Earth, permafrost plays a disproportionately large role in the global climate system. Consequently, robust and expansive monitoring of regions with changing permafrost will be essential through the 21st century.

The capacity for observing long‐term changes to permafrost and periglacial environments has gradually improved over several decades. The Circumpolar Active Layer Monitoring Network (CALM) was established in 1991 to observe long‐term, interannual impacts of variable climate on the active layer and near‐surface permafrost (Brown et al., 2000). The Global Terrestrial Network for Permafrost (GTN‐P), established by the International Permafrost Association in 1999, operates a comprehensive and long‐term global monitoring network of key permafrost physical parameters (Streletskiy et al., 2017). More recently, Global Navigation Satellite System (GNSS) reflectometry has been used to resolve both annual and inter‐annual surface deformation associated with thawing of the active layer (Hu et al., 2018; Liu & Larson, 2018). Although GNSS and dedicated in‐situ monitoring efforts like CALM can provide precise estimates of permafrost subsidence, these are point measurements that may not adequately represent permafrost changes away from the point of observation. The vastness of permafrost regions and the general inaccessibility of much of the northern high latitudes hamper many conventional methods of in situ monitoring. As a result, remote sensing techniques such as visible (e.g., Quinton et al., 2010) or multispectral (e.g., Nitze & Grosse, 2016) imagery mapping, lidar surveying (e.g., Jones et al., 2013), and synthetic aperture radar (SAR) analysis (e.g., Liu et al., 2010), have been employed to monitor permafrost, with varying degrees of success.

Interferometric synthetic aperture radar (InSAR) is a geodetic technique that can resolve centimetric deformation of the Earth's surface (e.g., Goldstein & Zebker, 1987; Rosen et al., 2000). InSAR has been successfully applied to study a range of phenomena in permafrost regions that give rise to surface deformation, including seasonal thawing of the active layer (Liu et al., 2012), wildfire‐induced thermokarst (Liu et al., 2014), initiation of retrogressive thaw slumps (Zwieback et al., 2018), and post‐wildfire active layer thaw and recovery (Michaelides et al., 2019). Although InSAR processing is capable of resolving deformation over vast spatial extents, precise estimates of deformation require several repeat observations and interferometric coherence from image to image. Extensive vegetation cover, changes in surface water cover, extent, and saturation, and variable snow cover, all of which are ubiquitous phenomena in permafrost regions, can induce signal decorrelation over temporal baselines as short as several weeks and limit the precision with which InSAR analysis can determine deformation in permafrost regions.

The September 2018 launch of the Ice, Cloud, and land Elevation Satellite 2 (ICESat‐2) mission (Markus et al., 2017) provides an opportunity to complement InSAR techniques with spaceborne laser altimetry that extends to ±88° latitude. The small footprint, fine‐scale along‐track spacing, and high precision of elevation retrievals (e.g., B. Smith et al., 2020) suggests that ICESat‐2 data products should be of sufficient quality to estimate surface deformation in complex permafrost terrain. Whereas C‐band InSAR decorrelates across temporal baselines longer than a few weeks, ICESat‐2 can yield long‐period temporal information without signal degradation. Similarly, InSAR and laser altimetry are sensitive to different atmospheric characteristics (radar is sensitive to tropospheric water content, whereas laser altimetry is sensitive to cloud cover), providing complementary observations of permafrost evolution and hazards in a challenging atmospheric environment.

In this work, we provide an initial assessment of the capability of the ICESat‐2 mission to quantify spatial patterns of surface‐height change in periglacial environments. We derive estimates of surface‐height change from ICESat‐2 crossovers and repeat transects over a region of the Alaskan North Slope near the 2007 Anaktuvuk River Fire scar. We demonstrate a linear relationship between ICESat‐2‐derived surface‐height change estimates from crossover analysis and changes in the normalized accumulated degree days (NADD), a proxy for differential thaw, which suggests that ICESat‐2 altimetry crossover‐derived height change is directly sensitive to the seasonal thaw and refreeze of the active layer. We also show that ICESat‐2 observations are sensitive to the depth of snow cover and apply a correction based on independent reanalysis data to remove this snow‐cover bias from ICESat‐2 repeat transects. Integrating ICESat‐2 crossover estimates of surface‐height change yields an annual, region‐wide time series of surface height, which exhibits temporal agreement with both an independent InSAR‐derived time series, as well as temperature and snow‐cover reanalysis datasets. We further find that ICESat‐2 estimates of surface‐height change are significantly impacted by short length‐scale (and sub‐footprint) topographic gradients, even for effectively overlapping (<5 m) data segments. This short length‐scale topographic bias can introduce surface‐height change biases on the order of, or larger, than expected deformation signatures and represents a major error source that must be corrected before a robust ICESat‐2 surface‐height change product over typically rough periglacial terrains can be generated. Finally, we discuss several possible methods of further refining surface‐height change estimates and suggest future steps for expanding ICESat‐2 data analysis to pan‐Arctic estimates of Arctic permafrost change.

## 2 Methods

### 2.1 Field Site

**Figure 1:**
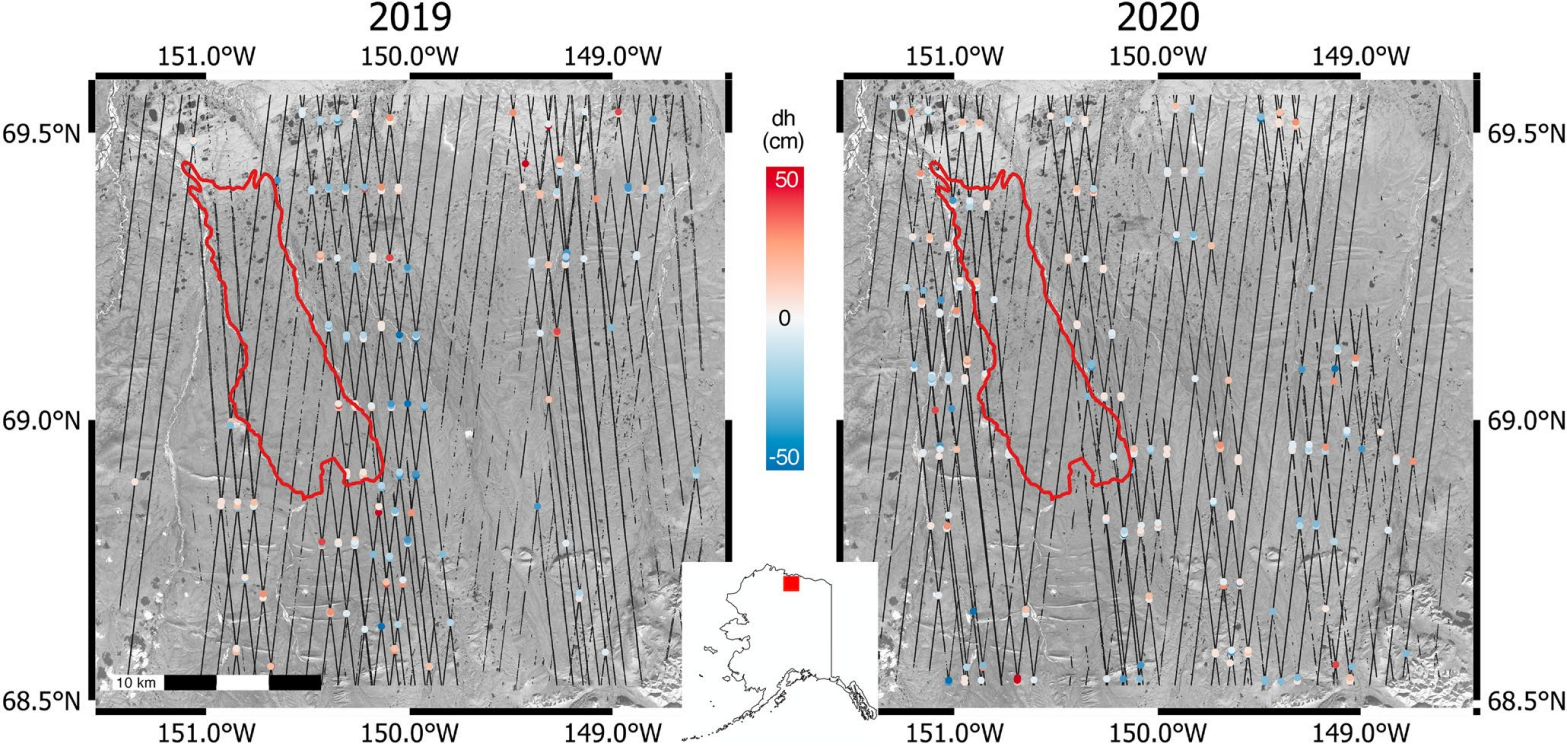
Study site within the Alaskan North Slope, with relative position within Alaska (inset). Left: 2019 ICESat‐2 crossovers. Right: 2020 ICESat‐2 crossovers. Anaktuvuk River Fire scar is outlined in red in both images. Background imagery source: EarthStar Geographics SIO.

We compared Sentinel‐1 InSAR deformation and ICESat‐2 ground surface‐height change in a 3,220 ${km}^{2}$ region of the North Slope of Alaska that encompasses the foothills of the Brooks Range to the south and the Arctic coastal plain to the north (Figure 1). Although the southern reaches of the study region exhibit considerable topographic relief, the tundra to the north of the foothills is flat and characterized by heath vegetation, tussock tundra, and wet sedge tundra along well‐drained hilltops, hillslopes, and saturated lowland valleys, respectively (J. Chen et al., 2020). Despite the lack of large‐scale topography to the north of the Brooks Range, the tundra is characterized by complex microtopography due to polygonal landforms, tussocks, and fine‐scale drainage features (Paine et al., 2013; Zona et al., 2011). Furthermore, the 2007 Anaktuvuk River Fire led to increased degradation of ice‐wedge polygons, the formation of new thermokarst features, and an increase in the roughness and complexity of areas of the tundra affected by the fire (Jones et al., 2015). The entirety of the Alaskan North Slope is underlain by continuous permafrost, with reported active layers ranging from 40 to 100 cm in depth (Brown et al., 2000).

Ignited by a lightning strike on July 16, 2007, the Anaktuvuk River Fire burned ∼1,039 ${km}^{2}$ of tundra in our study region, resulting in a doubling of the cumulative burned area of the Alaskan North Slope over the last 50 years (Jones et al., 2009) and a release of ∼2.1 Tg of carbon into the atmosphere—equivalent to the net annual carbon sink of the circumpolar Arctic tundra (Mack et al., 2011). Both field measurements and InSAR measurements have indicated post‐fire increases in active layer thickness and seasonal subsidence of the tundra burned by the Anaktuvuk River Fire (Liu et al., 2014; Rocha & Shaver, 2011).

**Figure 2:**
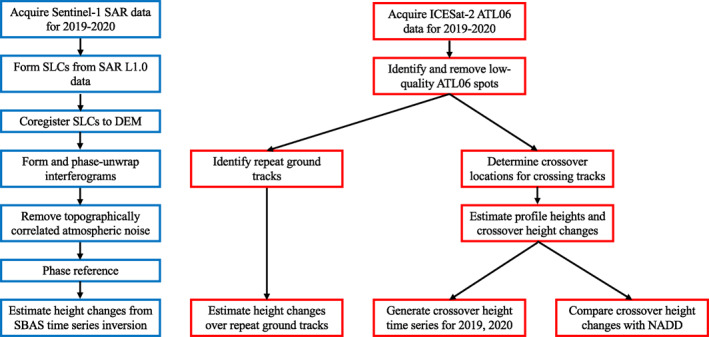
Data processing workflow for InSAR data (blue) and ICESat‐2 data (red).

The details of the processing methodology for both InSAR and ICESat‐2 datasets are expressed diagrammatically in Figure 2 and described in the following subsections.

### 2.2 InSAR

We used SAR data acquired between January 7, 2019 and 21 December 2019 by the Sentinel‐1A satellite, which operates at C‐band (∼5.65 cm wavelength) and has a 12‐day temporal repeat in the high Arctic. We processed raw data (L1.0) collected in the interferometric wide swath mode using the “geocoded single‐look complex” (SLC) backprojection method (Zebker & Zheng, 2016; Zheng & Zebker, 2017). All SLC radar images were coregistered to a digital elevation model (DEM) spanning the region of interest and were produced from the photogrammetric ArcticDEM data set (Porter et al., 2018). The DEM was downsampled to a resolution of ∼5 m by ∼15 m, to match the native 5 m by 15 m spatial resolution of the Sentinel‐1A satellite in range and azimuth, respectively.

We generated a network of interferograms from all coregistered SLCs using a temporal baseline of 24 days or less and a perpendicular baseline of 150 m or less. We took 18 looks in range and 6 looks in azimuth during interferogram formation to increase the signal‐to‐noise ratio (SNR) of the phase estimation, resulting in interferograms with a spatial resolution of ∼100 m in both range and azimuth. We then unwrapped all interferograms using the SNAPHU algorithm (C. W. Chen & Zebker, 2001). We used the correlation files of each interferogram to aid in the unwrapping scheme and tiled each interferogram to speed up computational time. We then applied a unimodal correction to all unwrapped interferograms to correct for any phase unwrapping errors in the unwrapped interferograms. All interferograms exhibiting severe decorrelation or turbulent atmospheric noise were removed from the set of interferograms used for analysis as in J. Chen et al. (2020). We removed the topography‐correlated component of atmospheric noise from all interferograms using the DEM following Zebker (2021). Due to the paucity of reliable GNSS stations in the study region, all interferograms were phase‐referenced using a selection of several pixels exhibiting high coherence in regions of no assumed deformation (i.e., mountain ridges) following Liu et al. (2012).

After applying the above calibrations to the InSAR data, we generated a pixel‐wise time series across the comparison region using the small baseline subset (SBAS) method over a network of 14 interferograms spanning the 2019 thaw season (Berardino et al., 2002). The SBAS method is an inversion that solves for the pixel‐by‐pixel instantaneous velocity at the time of each SAR image acquisition. We then integrated the estimated velocities through time to form a time‐series of surface displacements for each pixel over the temporal range of the network of input interferograms. A single interferogram with a 48‐day temporal baseline (August 11, 2019 to September 28, 2019) and exhibiting high coherence was included in the network of interferograms in order to restrict the network of interferograms to a single subset; all remaining interferograms consisted of 12‐day and 24‐day pairs.

### 2.3 ICESat‐2

ICESat‐2 is in a polar orbit with a 92° inclination, collecting observations from 88°N to 88°S with a 91‐day exact repeat (Markus et al., 2017). ICESat‐2's 532 nm laser instrument emits a single pulse, which is split into three pairs of beams, where each pair has one strong beam that is four times stronger than the corresponding weak beam. The three pairs are spaced at ∼3.3 km across‐track and beams within a pair are spaced at ∼90 m, with the exact geometry controlled by spacecraft attitude (Neumann et al., 2019).

Each beam illuminates a surface spot of 12–15 m diameter (Klotz et al., 2020) every 0.7 m along track. During the period covered by this study, ICESat‐2 operated in “mapping mode” away from polar regions, resulting in a higher density of tracks but no repeat measurements (Neumann et al., 2019). However, the North Slope of Alaska is a “target of opportunity” for the ICESat‐2 mission, where the satellite is off‐pointed from its regular pattern to observe a target of interest. In the case of the North Slope, every fifth descending track was repeated. This resulted in a small number of repeated tracks during the 2019 thaw season that is the focus of our study. We note that on September 9, 2019, the satellite performed a yaw flip in which the orientation of the altimeter instrument, and thus the relative ordering of weak and strong beams, was reversed.

We used surface‐height estimates from the Land Ice Height Product, ATL06 (B. E. Smith et al., 2019). The ATL06 algorithm processes, filters, and provides a linear fit to the geolocated surface photons along 50%‐overlapping 40 m segments to estimate the centroid height and surface slope in the along‐track and across‐track directions (B. Smith et al., 2019). We only used ATL06 data points flagged as high quality and that had a height within 2 m of adjacent segments. In addition, we removed segments with surface‐height uncertainty >1 m, along‐track slope > 0.05, and a signal‐to‐noise ratio significance level <0.02.

We estimated surface‐height changes from both repeated tracks and profile crossover points. To identify crossing locations, we divided the study area into 10 km latitudinal bands. Within each band, we fit lines to the longitude and latitude coordinates of ATL06 segments on individual tracks and calculated all intersections. For each crossover, we then constrained the data from the crossing tracks to segments lying within a specified radius of the crossing location. We considered the crossover valid if the track had a density of at least 1 point every 40 m, then recalculated the precise crossover location using these local segments. We estimated the profile heights at the crossover location using a line fit to ATL06 segment heights as a function of along‐track distance. Finally, we estimated the crossover height difference (dh) as the difference of the profile elevations at the crossover location, subtracting the later observation in time from the earlier observation. We filtered crossover dh results to remove estimates that were further than 2 standard deviations from the mean of the entire set of crossover dh estimates. We propagated uncertainties on individual ATL06 elevations through to the final dh estimates.

To examine the sensitivity of our crossover estimation to our choice of interpolation distance, we tested distances ranging from 20 to 100 m for fitting lines to ATL06 segments on either side of the crossover location. We used two metrics to assess the performance of a given interpolation distance. The first is the median propagated uncertainty for all crossovers calculated, which is a function of both the uncertainty of each ATL06 surface‐height estimate and of the residuals of the linear fit. The second is the standard deviation of crossovers with a time interval of 14 days or less, a period over which we assume surface‐height change is negligible.

To assess the ability of crossover estimates to characterize large‐scale subsidence patterns over time, we searched for crossovers over the entire time period of available ATL06 data, which spanned October 14, 2018 to November 10, 2020. We also examined the 2019 and 2020 thaw seasons individually, to assess the ability of crossover estimates to detect thaw‐season subsidence. When selecting the time interval over which to define the thaw season, we sought to limit the amount of snow‐cover change, while maintaining a sufficient number of crossovers for our analysis. To this end, we calculated the number of crossovers and relevant statistics in four different time intervals, using different data constraints. The first time interval corresponds to the date range of the interferograms used for the SBAS inversion. The second time interval starts with the onset of thaw of the active layer and ends with autumnal refreeze, corresponding to the time period over which the seasonal accumulated degree days (ADD) were positive. We calculated ADD from a daily time series of air temperature at 2 m height above the surface from NASA's Daily Surface Weather and Climatological Summaries (DAYMET) reanalysis temperature data set (Thornton et al., 2016). The third interval is the snow‐free period based on (Hall & Riggs, 2016). This period was defined as the time interval ranging from the first date in summer where there was no snow observed on the land surface, to the first date in autumn with visible snow. The fourth interval consists of the months where the monthly mean diurnal snow fraction (Global Modeling and Assimilation Office, 2015) was less than 5% across the study region (Gelaro et al., 2017).

To assess the degree to which crossover surface‐height change estimates are sensitive to the seasonal thawing (subsiding) and freezing (uplift) of the active layer, we assumed that surface deformation due to seasonal thaw and refreeze follows a Stefan's Law relationship; that is, deformation is proportional to the square root of the ADD between any two observation times (Hinkel & Nicholas, 1995; Nelson et al., 1997). We normalized the DAYMET‐derived ADD time series described above with respect to the maximum ADD value from each thaw/freeze season to remove dependence on the n‐factor, a source of uncertainty in quantifying absolute ADD (Liu et al., 2012). Normalized accumulated degree days (NADD) therefore range from 0 to 1. We calculated the change in the square root of NADD between each crossover pair, which we used as a proxy for the expected deformation of the ground surface due to freeze/thaw. In this manner, positive changes in the square root of NADD correspond to subsidence due to thaw, whereas negative changes correspond to uplift due to refreeze; the farther apart in time between the ICESat‐2 tracks in each crossover pair, the larger the corresponding magnitude of change in NADD.

We estimated annual time series of surface‐height change over the study site from crossovers for both the 2019 and 2020 seasons. We assumed that the surface moves uniformly everywhere throughout the study site and invert for the height change time series that best fits the crossover data in a least squares sense. For fitting, we used a functional form that minimized the sum of the residual between modeled and observed dh with constraints on model size (with weight $\lambda_{w}$) and smoothness (with weight $\lambda_{s}$). We estimated model uncertainty in two ways: (a) we propagated uncertainties in the individual crossovers using a Monte Carlo simulation with 200 realizations of the input crossovers, generated from the observed crossover dh by applying random Gaussian error scaled by the crossover standard deviation; and (b) we used a jackknife approach to uncertainty estimation, in which we discarded a random selection of up to 50% of the observed crossover values and re‐estimating the model since uneven spatial sampling of the study domain is another potential error source.

We identified potential repeat tracks by flagging tracks from the same Reference Ground Track (RGT) with different collection dates and corresponding beams that had an across‐track distance difference of <45 m. For each point from the earlier profile, we identified the closest point on the later profile and calculated the distance between observations. In order to ensure sufficiently overlapping footprints, we only kept pairs that were within 5 m of each other. We then calculated the surface‐height difference between each pair. Due to the low temporal and spatial frequency of repeats, we searched for repeats within the full SBAS time frame. Through this process we identified four RGTs with repeat profiles and consistent collection across the region of interest, including three 182‐day repeats and one 91‐day repeat. Six additional tracks had sparse coverage, likely due to cloud cover. We selected the 91‐day repeat track (RGT 1280) and one of the 182‐day repeats tracks (RGT 274) for direct comparison with the InSAR results. In order to reduce the noise in our final results, we applied a boxcar filter over 2 km. We estimated the change in snow depth between the two dates by differencing the MERRA‐2 mean monthly snow depths for each month and subtracting this value from our height‐change estimates.

## 3 Results

### 3.1 InSAR

**Figure 3:**
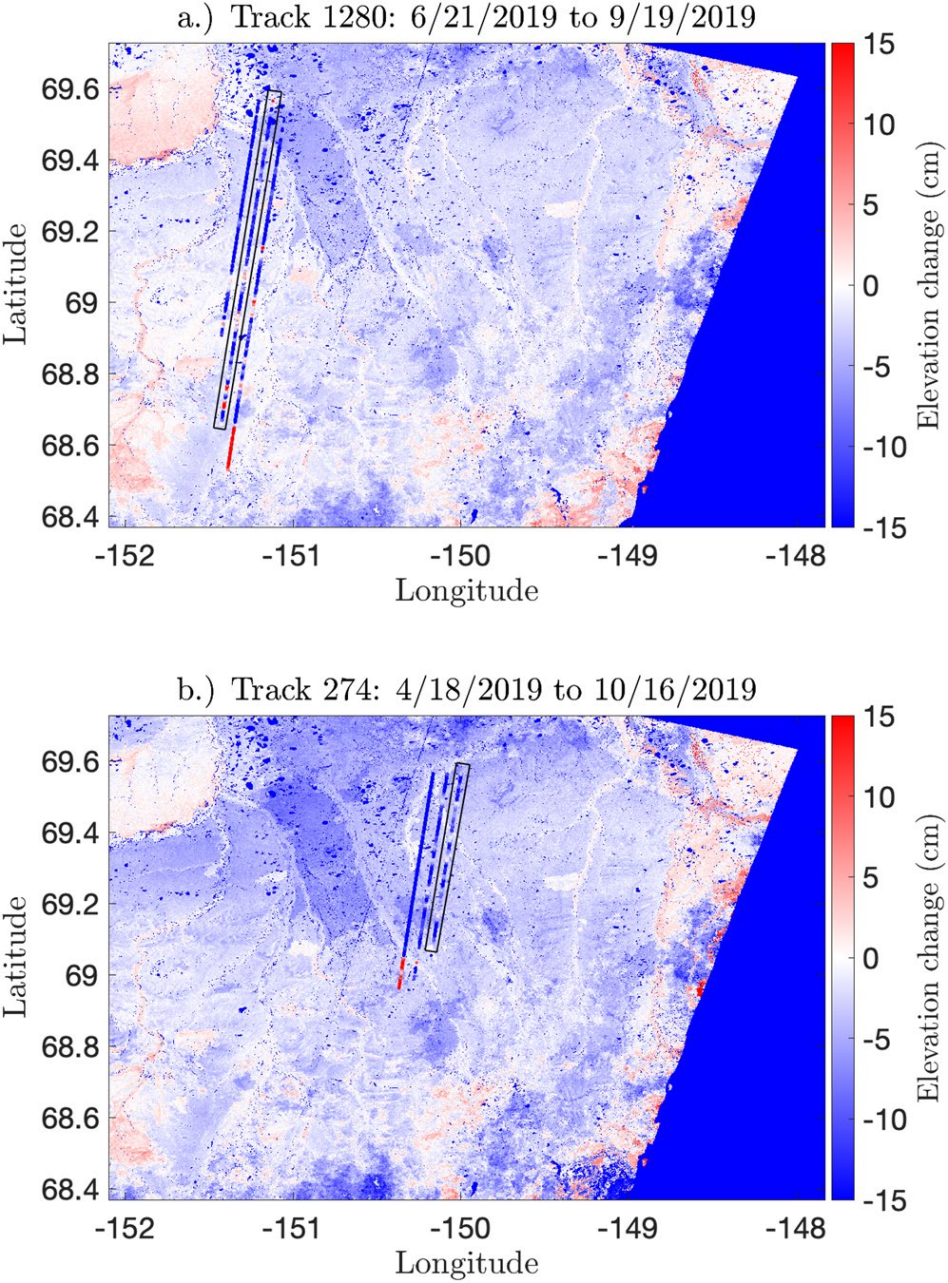
Comparison of the surface‐height change from 91‐day (RGT 1280) and 182‐day (RGT 0274) repeat tracks. (a) 91‐day surface‐height change and (b) 182‐day surface‐height change overlain over InSAR‐derived deformation estimates over similar time periods. The left beams of the pairs highlighted in black are shown in Figure 8.

We applied the SBAS algorithm to 14 interferograms spanning the 2019 thaw season. The SBAS algorithm solves for a time series of instantaneous velocity estimates for each epoch at which a SAR image was acquired. Integrating this velocity time series yields a time series of surface deformation, which can be directly compared to all deformation estimates derived from ICESat‐2 data. The SBAS method resolves increased subsidence over the 2007 Anaktuvuk River Fire scar (red outline, Figure 1). We observe a ∼1.5 cm difference in subsidence between the burned tundra and unburned tundra (Figure 3), which is consistent with estimates of 12 year post‐fire active layer recovery from the Yukon‐Kuskokwim delta (Michaelides et al., 2019).

### 3.2 ICESat‐2 Crossovers

**Figure 4:**
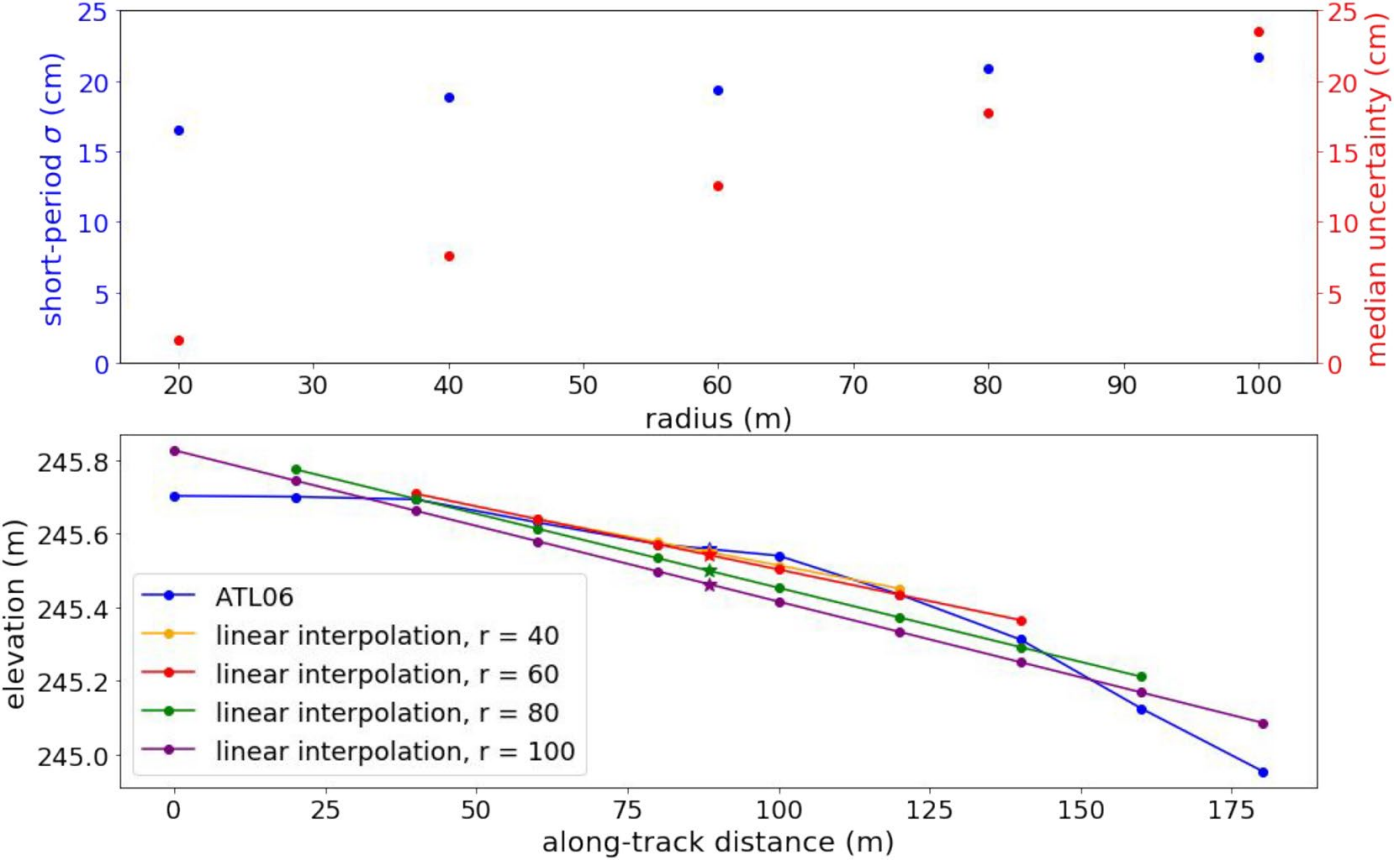
Top: standard deviation of short‐period (<14 days) crossovers (left axis) and median propagated uncertainty (right) as a function of interpolation radius. Bottom: an example of linear fitting of an ATL06 profile in this region, where the star represents the crossover location. As the interpolation radius increases, the interpolation does a poorer job of fitting the surface, and the crossover surface‐height estimate deviates further from the true surface.

For the bulk statistics reported in this work, we compared 1,018 individual ICESat‐2 beams from 185 ascending and descending tracks spanning the full ICESat‐2 study period, and identified 33,975 potential crossovers. From this set, there were between 13,728 and 16,021 crossovers with the required point density, depending on the interpolation radius. The crossovers spanned time periods ranging from 3 to 157 days, with 463 to 533 “short‐period” crossovers spanning 14 days or less. Figure 4 shows the standard deviation of short‐period crossovers and median propagated uncertainty (1σ) as a function of radius. The median uncertainty increases sharply with interpolation distance. When interpolating using only the nearest two points, the propagated uncertainty is a function of the uncertainty in each of the ATL06 segments. When interpolating over more than two points, the propagated uncertainty is also dependent on the residuals of the linear fit. Although we would expect interpolations over longer length scales to reduce the uncertainty for flat areas, the topography in this region is complex, leading to high residuals when interpolating over several ATL06 segments, which results in high propagated uncertainties. The short‐period standard deviation is a function of both cross‐track bias and the accuracy of the estimate of the elevation at the crossing location. In areas where the topography is non‐linear, such as the example shown in Figure 4, the accuracy of the elevation estimate at the crossing location decreases as the interpolation length increases. This in turn can decrease the accuracy of the height‐change estimate. Both the uncertainty and short‐period standard deviation are minimized for the 20 m interpolation, with a median uncertainty of 1.7 cm across all estimates and a standard deviation of 17 cm for the 463 short‐period crossovers. Therefore, we concluded that interpolation using only the nearest two points is the optimal solution given the terrain. There is the possibility that the topography is also non‐linear on length scales shorter than 20 m, but this issue cannot be directly addressed with ATL06. Accordingly, we only used crossovers between tracks with two points within 20 m of the crossing location for the subsequent analysis, yielding 13,728 crossovers over the full ICESat‐2 study period.

**Figure 5:**
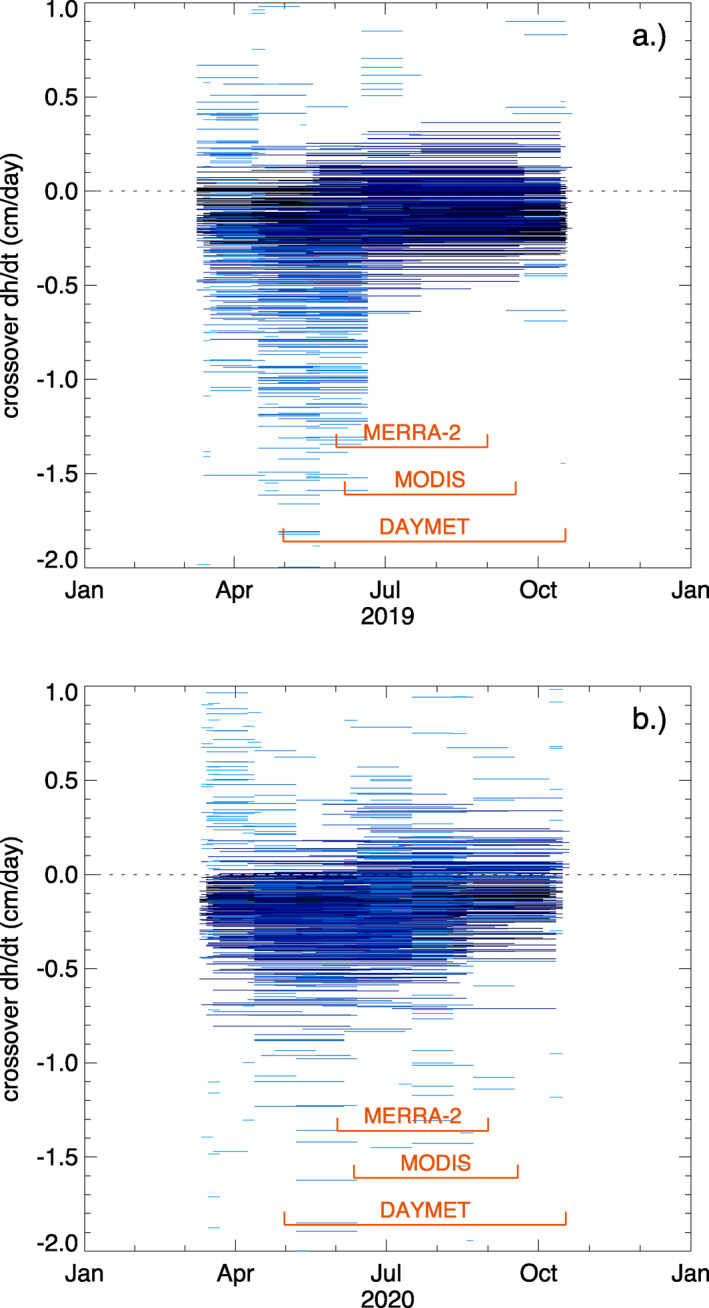
(a) 2019 crossover values and (b) 2020 crossover values normalized by time between first and second acquisition. Light blue crossovers correspond to shorter time intervals (as small as 3 days), whereas dark blue crossovers correspond to larger time intervals (up to 218 days). Thaw season time intervals as derived from MERRA‐2, MODIS, and DAYMET datasets are denoted with orange brackets.

Of the 13,728 available crossovers, 1,744 fall within one of the two potential thaw seasons as defined by the time interval of InSAR time series, with 805 in 2019 and 939 in 2020. Table 1 shows the date range of four time intervals considered for both the 2019 and 2020 thaw seasons and the corresponding crossovers statistics for each range, calculated for the case of a 20 m interpolation distance. The interferograms used span 8 March to 22 October. The ADD time series derived from DAYMET suggests a thaw period of 30 April to 16 September and refreeze period of 16 September to 17 October. Thus, we defined the full freeze‐thaw time interval as 30 April to 17 October. Since DAYMET estimates are not yet available for the summer of 2020, this window is applied to both years. Using the Terra/MODIS imagery, we identified 6 June as the first apparent snow‐free day of the summer and 17 September as the first day in the fall when snow re‐appears in 2019. For 2020, this interval was estimated to be from 11 June to 18 September. Based on MERRA‐2, the months with low snow coverage (less than 5% snow fraction) are June, July, and August for both 2019 and 2020. We also calculated the number of crossovers that corresponded to an increase in ADD ($n_{thaw}$) or a decrease in ADD ($n_{freeze}$). For the interferogram‐based range, crossovers that occurred entirely before thaw or after refreeze, and consequently have a zero change in ADD, were not counted in either $n_{thaw}$ or $n_{freeze}$. By scaling each crossover dh estimate by the time interval, we generated a time series of the surface‐height change, dh/dt, for 2019 and 2020 (Figure 5).

**Table 1:** Summary Statistics for the Crossovers Calculated Using a 20 m Search Radius for Four Different Time Intervals Based Upon Different Data Constrain Criteria

Year	Criteria	Date range	Total crossovers: $n_{thaw}$, $n_{freeze}$	Mean dh (cm)	Range (cm)	σ (cm)	Short‐period crossovers
2019	Interferograms	3/08 – 10/22	805: 483, 103	−15.1	−60.5 – 52.6	22.0	79
2020	Interferograms	3/08 – 10/22	939: 701, 135	−11.6	−60.3 – 52.0	20.9	141
2019	DAYMET	4/30 – 10/17	241: 191, 50	−4.9	−60.4 – 52.6	20.5	23
2020	DAYMET	4/30 – 10/17	400: 318, 82	−5.5	−59.5 – 47.2	18.7	71
2019	MODIS	6/06 – 9/17	35: 35, 0	6.2	−23.6 – 43.2	15.8	4
2020	MODIS	6/11 – 9/18	188: 188, 0	−2.5	−52.5 – 47.2	15.2	33
2019	MERRA‐2	6/01 – 8/31	30: 30, 0	8.9	−23.6 – 43.2	15.0	4
2020	MERRA‐2	6/01 – 8/31	185: 185, 0	−2.7	−52.5 – 47.2	15.2	41

**Figure 6:**
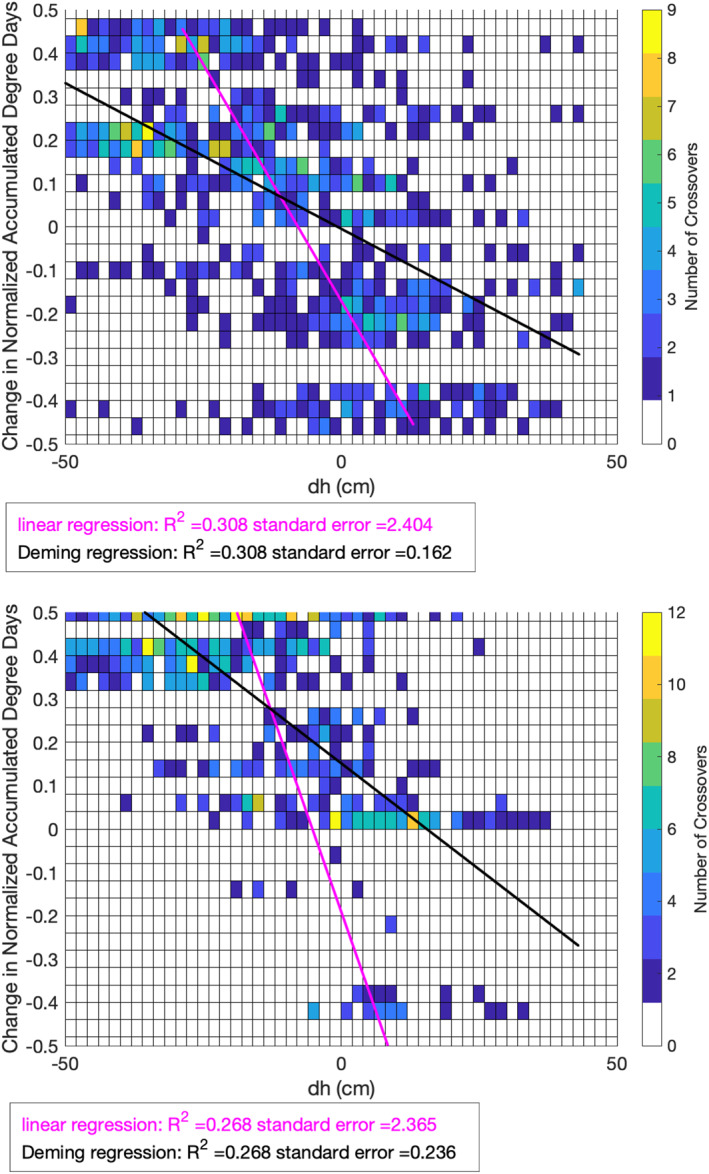
Two‐dimensional histogram of ICESat‐2 crossover‐derived surface‐height change estimates compared to the associated change in normalized accumulated degree days (NADD), with the results from a linear regression (magenta) and Deming regression (black) overlain.

The large spread in the crossover‐derived surface‐height change estimates (as indicated by the short‐period crossovers) complicated any direct analysis of individual crossovers. Instead, we performed a regional, spatiotemporal analysis of all crossover‐derived surface‐height changes. Plotting a two‐dimensional density plot between ICESat‐2‐derived vertical deformation of the ground surface and change in the square root of NADD reveals a clear temporal relationship ($R^{2}$ = 0.308 for 2019, $R^{2}$ = 0.268 for 2020). We performed a linear regression and a Deming regression–a total least squares method which accommodates errors in both variables in the regression. Both regressions resulted in statistically significant linear relationships between surface‐height change and change in NADD (p<0.001), although accounting for uncertainties in surface‐height in the Deming regression (λ=0.01) resulted in a significant reduction in the standard error of the regression (Figure 6). The magnitude of ICESat‐2‐derived vertical surface deformation is positively correlated with the magnitude of NADD change, whereas the sign of ICESat‐2‐derived vertical surface deformation is negatively correlated with the sign of NADD change. This relationship is physically consistent with active layer thawing (i.e., subsidence) during time spans where degree days accumulate–warming summer months–and freeze‐up (i.e., uplift) during active layer freezing, when changes in degree days are negative. Although the relationship between surface‐height change and change in NADD is statistically significant, there is still statistical variance unaccounted for by both regressions, likely due to a combination of residual snow cover, spatial heterogeneity, residual errors due to crossover interpolation, and topographic gradient‐correlated errors.

**Figure 7:**
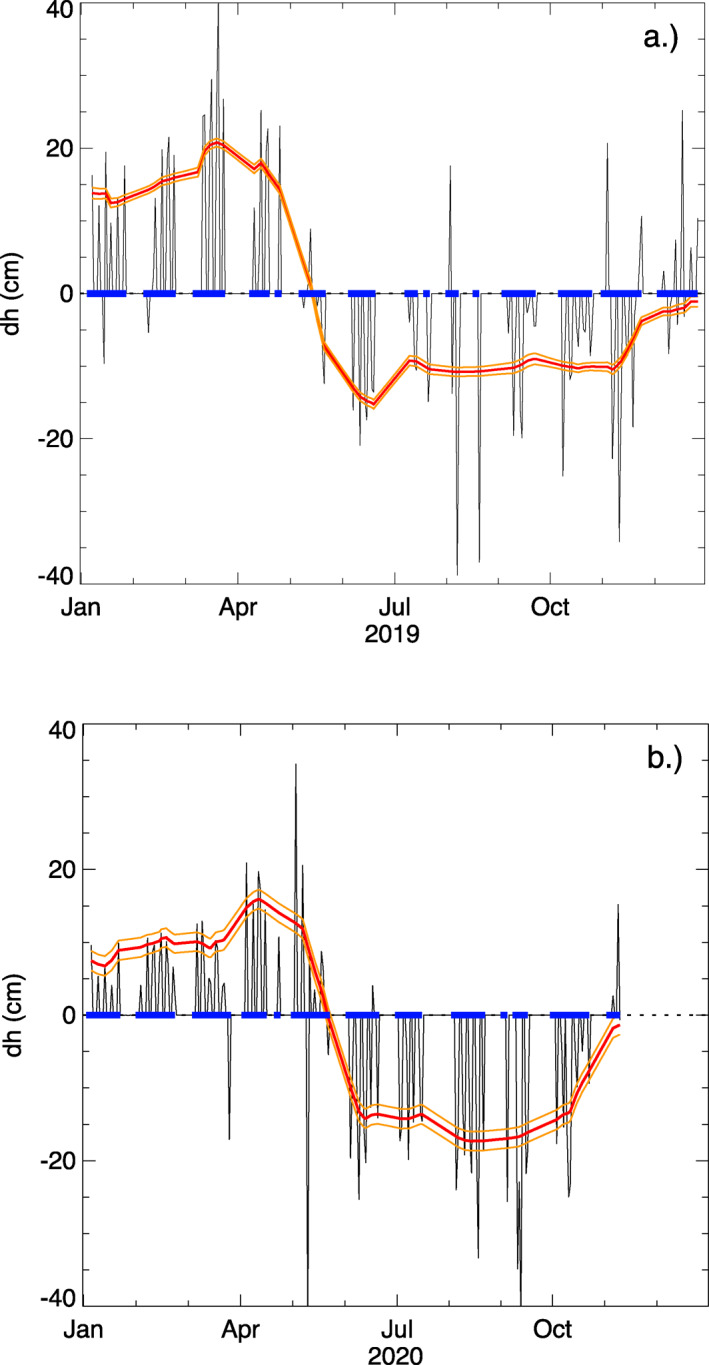
(a) 2019 times series and (b) 2020 time series model (red) derived from crossovers (black), with1−σ uncertainties (orange).

We derived a crossover‐based deformation time series model h(t) to explain observed crossover heights dh in terms of uniform changes in surface‐height across the study area. Our method used least‐squares inversion to minimize the residual between expected and observed crossover differences, initially implementing no smoothing and a minimal size constraint obtained by increasing the weight of the size regularization term in small increments from 0 until the shape of the model stabilized (black line, Figure 7). The times of the crossover endpoints are denoted by small blue boxes on the *dh* = 0 line, which is where this initial inversion places changes in the deformation time series. Although this model fits the observed data, it is aphysical in the sense that the surface does not instantaneously oscillate between zero and non‐zero values. We obtained a physically plausible model of surface‐height change (red line, Figure 7) by applying an additional smoothing constraint, choosing a weight for the smoothing regularization term that minimized sharp breaks in slope between crossover observations without damping long‐period changes in h(t). This selection criterion is qualitative, but we found that using the traditional method of choosing the weight from the point of maximum curvature of the model smoothness versus model residual function (the L‐curve method) yielded models that were too rough to be plausible. Monte Carlo uncertainty estimation yielded uncertainties that were several orders of magnitude smaller than the model value itself and were not visible when plotted. Thus, uncertainties were calculated using the jackknife approach; the standard deviation we obtained from 200 model realizations with 50% data sampling is the basis for the orange 1σ uncertainty lines shown in Figure 7.

The direct sensitivity of ICESat‐2 height‐change estimates to snow‐depth change is evident in both crossover dh/dt values (Figure 5) and the crossover‐derived time series of surface‐height change (Figure 7). Large dh values in early spring and large, negative dh/dt values in late spring are consistent with snow packs of ∼15 cm that do not melt until air temperatures are consistently above zero starting in May. Snow‐free conditions arise in May and early June, after which thawing of the active layer leads to subsidence of the ground surface throughout summer and into early autumn. The transition from snow‐covered to snow‐free conditions, and the associated decrease in surface‐height rates of change as rapid snow melt gives way to gradual thaw subsidence, is particularly evident in 2019 (Figure 5a).

### 3.3 ICESat‐2 Repeat Tracks

**Figure 8:**
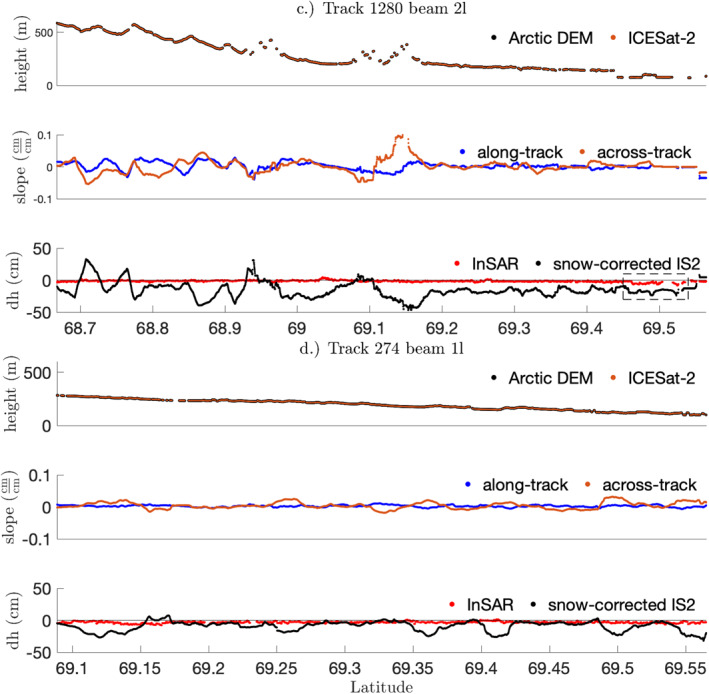
Comparison of the surface‐height change from 91‐day (RGT 1280) and 182‐day (RGT 0274) repeat tracks. (a) 91‐day and (b) 182‐day along‐track profiles. For both transects, we show surface‐height as derived by ArcticDEM and ICESat‐2 (top), ICESat‐2 along‐track and across‐track slopes (middle), and InSAR‐derived and snow‐corrected ICESat‐2‐derived estimates of surface‐height change (bottom). The dashed line box indicates the geographic bounds of the Anaktuvuk River Fire scar. Note the correlation between the topographic gradient (along‐track and across‐track slopes) and the ICESat‐2 surface‐height change estimates, particularly in Track 1,280.

The 10 total repeat tracks in this study yielded surface‐height changes ranging from −315 to 329 cm, with uncertainties between 0.8 and 95 cm. Overall, repeat‐track comparisons showed net subsidence over the study region, with a mean of −9.8 cm. As expected, the magnitude of height changes from 182‐day repeat tracks were higher (−315 cm <dh< 328 cm; 1.5 cm <σ< 95 cm) than that for the 91‐day repeats (−274 cm <dh< 218 cm; 0.81 cm <σ< 95 cm). We selected one 91‐day repeat (RGT 1280) and one 182‐day repeat (RGT 0274) for comparison to the InSAR results. Applying the 2 km boxcar filter reduced both the variance and the propagated uncertainty in the data. For RGT 0274 spot 1L, the smoothing filter reduced the standard deviation and median uncertainty from 19 and 3.2 cm to 7.8 and 0.4 cm, respectively. For track 1280 spot 2L, the smoothing filter reduced the standard deviation and median uncertainty from 21 and 6.4 cm to 12 and 1.3 cm. The spatial distribution of the averaged dh values after removing the snow signal for each of the two RGTs is shown in Figure 8.

To validate ICESat‐2 surface‐height change estimates from repeat tracks, we compared our ICESat‐2 surface‐height change estimates to SBAS‐derived deformation over approximately the same temporal baseline (Figure 3). We compared SBAS‐derived deformation observed between dates June 23, 2019, and September 16, 2019, with ICESat‐2‐derived 91‐day height change from RGT 1280, which spanned June 21, 2019, and September 19, 2019 (Figure 8a), as well as SBAS deformation between April 20, 2019, and October 23, 2019, and ICESat‐2‐derived 182‐day height change on RGT 0274, which spanned April 18, 2019, and October 16, 2019 (Figure 8b). The difference in acquisition date between the first ICESat‐2 observation and Sentinel images and second ICESat‐2 observation and Sentinel images is 3 days for RGT 1280 and 5–6 days for RGT 0274, such that the expected deformation of the surface between the inter‐instrument image acquisition can be assumed small. As such, the surface deformation observed by InSAR and ICESat‐2 are expected to be roughly equivalent.

Both the spatial pattern and sign of observed surface‐height changes (i.e., uplift or subsidence) were broadly consistent with InSAR observations on flat terrain (Figure 3). However, the magnitude of surface‐height change calculated from ICESat‐2 data was often much higher than that estimated from InSAR observations, largely due to nonzero topographic gradients in the along‐track and across‐track directions. A two‐sample Kolmogorov‐Smirnov test rejected the null hypothesis that ICESat‐2 transect‐derived surface‐height changes over the Anaktuvuk River Fire are derived from the same probability distribution function as surface‐height change estimates over unburned tundra (p<0.001), which suggests that ICESat‐2 is directly sensitive to either differences in surface scattering properties or differential deformation patterns between burned and unburned tundra. Additionally, ICESat‐2 surface‐height changes displayed a moderate correlation with InSAR‐derived surface‐height changes within the Anaktuvuk River Fire scar ($R^{2}$ = 0.194), whereas the correlation between these two measurements outside the fire scar is lower ($R^{2}$ = 0.014). When comparing the sampled distribution of transect‐derived surface‐height changes between the burned and surrounding unburned tundra (as measured between 69.19° N and 69.45° N), we note that the ICESat‐2 surface‐height changes display a lower standard deviation in the burned tundra (σ=3 cm) compared to the unburned tundra (σ=5.5 cm). However the mean subsidence is approximately equal in both regions (dh=17 cm).

Prior to correcting for snow depth, the magnitude of deformation from the 182‐day repeat was systematically higher than those from the 91‐day repeat. MERRA‐2 data indicated a decrease in snow depth of 27–32 cm in the area between April and October. After removing this change in snow depth, the corrected dh estimates were much closer to the InSAR‐based estimates, and the areas where they diverge appeared to correspond with high along‐track slopes (Figure 8). However, applying snow corrections to the 91‐day repeat did not improve the agreement with InSAR estimates. The MERRA‐2 data suggested that there was an increase in snow depth of 6–9 cm due to snow in late October, so applying this correction increased the magnitude of our dh estimates. MERRA‐2 provides a monthly mean, so the timing of snow accumulation with respect to ICESat‐2 observations is uncertain. Although a monthly data set such as MERRA‐2 may be useful for longer period (two or more orbital cycles) repeat tracks, higher resolution snow‐depth estimates are needed to understand the effect of snow cover on ICESat‐2 elevation‐change measurements at shorter timescales. An increase in the number of repeat tracks could also increase the likelihood of sampling a snow‐free interval over the thaw season, from which higher fidelity estimates of seasonal subsidence of the active layer can be derived.

## 4 Discussion

Sentinel‐1 InSAR, ICESat‐2 crossovers, and ICESat‐2 repeat tracks capture spatially consistent large‐scale patterns of surface deformation associated with subsidence of the thawing active layer. However, ICESat‐2‐derived observations are systematically noisier than InSAR measurements, requiring spatial filtering for comparison with InSAR measurements. In the case of crossovers, performing a regional‐scale spatial analysis of surface‐height change reveals a clear correlation between surface‐height change and NADD, consistent with snow melt and thermodynamically driven subsidence of the active layer. We mitigated errors in ICESat‐2 transects through an along‐track filter with a boxcar impulse response 100 segments (2 km) wide, yielding comparable deformation estimates to the InSAR results. Further, the sensitivity of ICESat‐2 altimetry to snow cover, although an important geophysical signal itself, can significantly degrade surface‐height change estimates without empirical corrections derived from independent datasets or models, such as MERRA‐2.

Whereas InSAR‐derived estimates of surface‐height change rarely exceed 5 cm, surface‐height change estimates from ICESat‐2 repeat tracks approach 50 cm of uplift or subsidence—values that are inconsistent with the seasonal freezing and thawing of the active layer. Rather, these large inferred surface‐height change estimates appear to be systematic biases related to nonzero across and along‐track topographic gradients. For RGT 1280 2L, we find a moderate correlation between dh and along‐track slope ($R^{2}$ = 0.308) and strong correlation between dh and across‐track slope ($R^{2}$ = 0.578). For RGT 274, we also find a weak correlation with along‐track slope ($R^{2}$ = 0.09) and a strong correlation with across‐track slope ($R^{2}$ = 0.624) (Figure 8). In areas with relatively steep slopes, even small horizontal offsets between repeat measurements could result in a significant topographic contributions to height‐change estimates. In order to estimate the topographic contribution, we sampled the native‐resolution 2 m ArcticDEM at each point used for the repeat difference calculation using a bicubic interpolation of the nearest 16 DEM values. We then calculated the difference between the sampled elevations at the two dates. We find a weak correlation between these DEM differences for RGT 1280 2L ($R^{2}$ = 0.026) and a moderate correlation for RGT 0274 1L ($R^{2}$ = 0.325). This observation further reinforces the hypothesis of a topographic‐gradient bias, but a 2 m DEM may be insufficient to quantify this effect, due to the small size (<5 m) of typical ICESat‐2 misregistration offsets (Magruder et al., 2020). Furthermore, the geolocation uncertainty of an ATL06 segment in this region is estimated to be in the range of 2.5–4.4 m, based on a previous comparison with the 10 m Arctic DEM product (Luthcke et al., 2021). This limits our ability to correct for short length‐scale offsets. Finally, the possibility of non‐linear topography at the sub‐40 m scale and any changes in micro‐topography between repeats can all contribute to biasing surface‐height change estimates from ICESat‐2 repeat tracks.

The inherent discrepancy in measurement precision between InSAR and ICESat‐2 may be partially due to both the operational nature of the two imaging techniques, as well as their respective post‐processing methods. Although synthetic aperture radar interferometry and laser altimetry are both coherent source imaging techniques, the physical nature of each instrument's backscattered signal is different. SAR backscatter represents a convolution of the output radar signal with the distribution of scattering elements contained within each ground resolution element (resel). The distribution of scattering elements within any one individual resel may result in a noisy phase estimate, but considerable spatial averaging (“multilooking”) in both the along‐track and across‐track of the radar image results in a larger signal‐to‐noise ratio (SNR) and more precise phase estimate (Goldstein et al., 1988; Li & Goldstein, 1990; Zebker & Villasenor, 1992). Starting from SAR images with a native resolution of ∼5 m by ∼15 m across‐track and along‐track, respectively, a total of 18 looks across‐track and 6 looks along‐track were taken during interferogram image formation to generate images with a 100 m spatial resolution in both along‐track and cross‐track. As such, each individual phase estimate represents a statistical average of 108 independent measurements. In contrast, the ICESat‐2 ATL06 data set has a native along‐track resolution of 40 m. The altimetric return from the ICESat‐2 laser is dictated by the count density of backscattered photons, which is typically <10 signal photons out of 200 trillion transmitted photons (Neumann et al., 2019). Although the deformation uncertainty of a native resolution InSAR pixel is on the order of 1 cm, ATL06 surface‐height estimates have a precision of 9 cm for best‐case targets (i.e., high reflectivity and low roughness) (Brunt et al., 2019), and any inferred deformation (i.e., change in height) will be even larger. Therefore, an even greater number of statistical averages would be necessary to achieve height‐change estimates from ICESat‐2 with the precision of InSAR methods. However, given the observed spatial heterogeneity of tundra surface topography and observed height change from ICESat‐2, increasing the precision of ICESat‐2 elevation change measurements to the order of InSAR measurements by incoherent statistical averaging may not be feasible in practice.

Moreover, because ICESat‐2 transects provide a one‐dimensional, along‐track measurement rather than a two‐dimensional image like SAR, achieving a comparable number of statistical samples as a 100 m InSAR pixel necessitates boxcar‐filtering ICESat‐2 data in the along‐track direction with a spatial resolution of ∼2 km. As such, InSAR and ICESat‐2 estimates of deformation will agree better in flatter regions such as the northern Arctic coastal plain, whereas topographically rough areas like the Brooks Range foothills exhibit larger differences in inferred deformation. Therefore, a large amount of along‐track filtering over complicated topography challenges assumptions of signal ergodicity and may result in biased estimates of deformation with large uncertainties. The ATL06 data product was designed primarily for surface slopes of 1° or less (B. Smith et al., 2019), whereas slopes in this region are often a few degrees or more. We only included height changes for areas with along‐track surface slopes less than 5°; however, stricter surface slope restrictions may be needed. Alternatively, uncertainties in ICESat‐2‐derived deformations could be reduced by adaptively varying the crossover interpolation and along‐track smoothing based on local topography. Employing these strategies could help refine ATL06‐based height‐change estimates in complex terrain, but there is also the possibility of sub‐40 m topographic variations that limit the accuracy of ATL06 segment heights. In this case, a higher‐level surface‐height data product derived directly from the geolocated photons that considers the unique topographic and roughness characteristics of permafrost regions would improve ICESat‐2's utility for long‐term thaw monitoring.

ICESat‐2 observations can retrieve estimates of surface deformation that are consistent with independent InSAR estimates, but achieving this result requires appropriate statistical averaging and is expected to work better in regions exhibiting more uniform surface topography and permafrost distribution at the km spatial scale. In regions that exhibit a large degree of spatial heterogeneity, the assumptions of signal ergodicity inherent to any statistical averaging techniques break down, and biases in estimated deformation can manifest (Michaelides, 2020; Zwieback & Meyer, 2020). The presence of significant vegetation cover and the complex roughness characteristics of tundra terrains—particularly tussock tundra—can introduce uncertainties in the ICESat‐2 height retrieval itself, as photons may backscatter from vegetation scattering elements distributed over several decimeters. The smaller native precision of interferometric measurements, as well as the two‐dimensional nature of InSAR images, makes InSAR measurements more robust to spatial variability than ICESat‐2. Nonetheless, we have demonstrated a sensitivity of ICESat‐2 to local‐scale deformation associated with seasonal thawing of the active layer, which necessitates future investigation into the full potential of ICESat‐2 observations for characterization of permafrost surface dynamics.

Consideration should be given to the differing roles of crossovers and repeat tracks. Crossovers can provide a large spatial and temporal distribution of surface‐height change measurements, particularly in areas where ICESat‐2 is operating in mapping mode. Our regional, spatiotemporal analysis of crossovers reveals that crossover‐derived surface‐height changes are sensitive to the seasonal freeze/thaw dynamics of the active layer and can be used to generate regional‐scale time series of ground subsidence due to active layer thaw. However, the success of such an analysis is dependent upon a sufficient number of crossovers from which statistically significant relationships can be inferred, and crossover surface‐height change estimates can be significantly biased by snow cover. ICESat‐2 repeat tracks provide profiles of along‐track surface‐height changes over seasonal to yearly time scales. This can be useful for investigating deformation of specific features, at potentially a higher spatial resolution than most multilooked InSAR deformation products. An important consideration for future ICESat‐2 operations is the trade‐off between crossovers and repeat track coverage: increasing the number of repeated tracks in the Arctic will increase the spatial coverage of repeats while reducing the spatial coverage of crossovers.

Future work is needed to explore the full complementarity of ICESat‐2 and InSAR datasets over periglacial regions. Whereas ICESat‐2 height estimates are sensitive to snow‐cover depth, InSAR signals often decorrelate in the presence of snow. Coherent surface‐height change estimates from 182‐day repeat tracks can provide a complementary data set to InSAR‐based studies. The direct sensitivity of ICESat‐2 observations to snow cover and snow‐cover depth changes is itself an important geophysical signal, which may require periglacial‐specific methods that account for the significant deformation exhibited by the ground surface through the year. Whereas the sidelooking viewing geometry of conventional SAR imaging systems makes them insensitive to surface water bodies, the nadir geometry of ICESat‐2 allows for precise estimates of surface water height levels and changes (Cooley et al., 2021; Ryan et al., 2020). Such measurements, combined with InSAR‐based measurements of surface subsidence and active layer thickness, would then allow for novel investigations of the spatiotemporal relationships between permafrost thaw, water table, and lake/river level heights, as well as potentially the horizontal flow of groundwater through the permeable active layer.

## 5 Conclusion

This study provides a preliminary investigation into the effectiveness of using ICESat‐2 ground surface‐height changes to study thaw subsidence of the active layer in a periglacial environment. We compared InSAR deformation estimates of the ground surface to ICESat‐2‐derived surface‐height changes from crossovers and 91‐day and 182‐day transect intervals from 2019–2020 across a region of the Alaskan North Slope.

Both crossovers and repeat tracks are capable of detecting large‐scale subsidence patterns over the thaw season, although additional repeat‐track data collection is necessary to better assess the short length‐scale noise characteristics of ICESat‐2 altimetry over periglacial terrains. The magnitude of crossover‐derived surface‐height changes exhibited a linear relationship with the change in normalized accumulated degree days. Further, we integrated crossover surface‐height changes into a regional‐scale time series of surface‐height change over the years 2019 and 2020, capturing both changes in snow‐cover depth in spring, snow melt in late spring, and subsidence of the thawing active layer through summer and early autumn. Analysis of ICESat‐2 repeat tracks revealed a clear sensitivity of the ATL06 product to snow cover and snow‐cover depth; removal of the snow‐cover depth bias from independent estimates of snow‐cover depth from MERRA‐2 yielded a closer correspondence between ICESat‐2 and InSAR estimates of surface‐height change over the 91‐day and 182‐day transects. ICESat‐2 surface‐height change estimates appear to be impacted by a significant bias that is correlated with the along‐track topographic gradient; accurate characterization of this bias term and its removal from ICESat‐2 crossovers and repeat tracks represents a major outstanding step towards the development of a dedicated technique of estimating surface‐height changes over periglacial terrains characterized by topographic complexity. Finally, we have demonstrated that ICESat‐2 is sensitive to changes in surface scattering properties associated with tundra wildfire events, although future work is needed to fully understand the relevant physical scattering mechanisms.

It may be possible to further refine ICESat‐2‐derived estimates of surface‐height change by limiting analysis to topographically smoother areas, such as the Arctic Coastal Plain to the north of our study site or by developing adaptive algorithms that account for more local topography variations during statistical averaging. Further investigation into the fundamental nature of the scattering physics which gives rise to radar and photon backscatter over tundra terrain is also warranted. Given the importance of permafrost dynamics to the global carbon cycle, we advocate for investigation into the full potential of using ICESat‐2 data products to quantify surface dynamics in permafrost and periglacial environments.

## Data Availability

Copernicus Sentinel data collected in 2019 was retrieved from ASF DAAC on 6 July 2020, processed by ESA. ICESat‐2 data are available via NSIDC (https://nsidc.org/data/atl06). DEMs provided by the Polar Geospatial Center under NSF OPP awards 1043681, 1559691 and 1542736.
